# Novel biallelic *TK2* mutations cause mitochondrial DNA depletion syndrome with infantile early-onset lipid storage myopathy

**DOI:** 10.1186/s13023-025-03639-x

**Published:** 2025-03-17

**Authors:** Duoling Li, Yixin Shi, Hanhan Sun, Chuanzhu Yan, Yan Lin

**Affiliations:** Department of Neurology, Shandong Key Laboratory of Mitochondrial Medicine and Rare Diseases, Research Institute of Neuromuscular and Neurodegenerative Diseases, Qilu Hospital of Shandong University, No. 107 West Wenhua Road, Jinan, 250012 Shandong China

**Keywords:** TK2, MDS, Lipid storage myopathy, Early-onset

## Abstract

**Background:**

Mutations in the *TK2* gene are strongly associated with mitochondrial DNA depletion syndrome (MDS), a severe condition with high mortality and poor outcomes. Although many MDS cases are reported, those linked to *TK2* mutations with lipid deposition are rare. Large deletions in the *TK2* gene are even rarer.

**Methods:**

We conducted whole-exome sequencing to find the gene linked to MDS, followed by genomic and structural analyses, histopathological, and functional analyses to assess the mutations' pathogenicity. Additionally, a HEK293T cell model with *TK2* mutations was created to investigate the impact of large deletions on mitochondrial function.

**Results:**

The patient was found to have a novel compound heterozygous mutation in the *TK2* gene, consisting of a large deletion spanning exons 5–10 (E5-E10 del) and a previously reported missense mutation (c.311C > A, p.Arg104His). Analysis of the patient's muscle tissue demonstrated a marked reduction in mtDNA content and a significant impairment in overall mitochondrial function. In the HEK293T cell model, the group with the deletion mutation exhibited a notable reduction in TK2 protein expression and levels of mitochondrial complex subunits when compared to the control group. Furthermore, there was an observed increase in ROS levels, a decrease in ATP production, and compromised mitochondrial respiratory chain function. Moreover, we conducted a comprehensive review of the previously reported genotypic and phenotypic spectrum of *TK2* mutations in the literature.

**Conclusions:**

This case report underscores the detrimental impact of large fragment deletion mutations in the *TK2* gene and elucidates their role in the pathogenesis of MDS. It broadens the spectrum of known *TK2* mutations and enhances our understanding of the structural and functional consequences of these mutations.

**Supplementary Information:**

The online version contains supplementary material available at 10.1186/s13023-025-03639-x.

## Introduction

Mitochondrial DNA depletion syndrome (MDS) is a collection of autosomal recessive inherited disorders characterized by an early onset in infancy and a rapid progression leading to premature death [1–3]. The most common molecular abnormality associated with MDS is a significant reduction in the number of copies of mtDNA, typically falling below 30% [4]. This decrease in mtDNA can result in mitochondrial dysfunction, impacting various tissues such as skeletal muscle, liver, and brain, leading to a range of systemic or tissue-specific manifestations [4, 5].

MDS has been linked to mutations in 8 genes, including thymidine kinase 2 (*TK2*), deoxyguanosine kinase (*DGUOK*), polymerase gamma (*POLG*), ATP-dependent and GTP-dependent succinyl coenzyme A synthetase ligases (*SUCLA2* and *SUCLG1*), *MPV17*, mitochondrial twinkle helicase, and the cytoplasmic p53-inducible small subunit of ribonucleotide reductase *(RRM2B*), have been implicated in the pathogenesis of MDS [5, 6]. Notably, mutations in *TK2* are predominantly associated with the myopathic variant of MDS [5, 7–9].

Mammalian cells possess two distinct thymidine kinases, TK1 and TK2, which catalyze the phosphorylation of thymidine (dThd) and 2′-deoxyuridine (dUrd) to their monophosphate forms [10]. Notably, TK2 serves as the exclusive pyrimidine nucleoside salvage enzyme in quiescent tissues such as skeletal muscle [11, 12]. Encoded by a nuclear gene located on chromosome 16 and targeted to the mitochondria, TK2 plays a critical role in the biosynthesis of DNA precursors necessary for mitochondrial DNA replication and in regulating the levels of deoxyribonucleotide triphosphate pools [13–15]. Disruption of these pools can lead to mutations or deletions in newly synthesized nuclear DNA or mitochondrial DNA, both of which encode essential components of the mitochondrial respiratory chain.

In this study, we document a case of an infant diagnosed with MDS, presenting as infantile early-onset lipid storage myopathy. This condition was caused by biallelic mutations in the *TK2* gene, including a large deletion spanning exons 5 through 10 and a missense mutation, c.311G > A (p.Arg104His). To elucidate the pathogenic implications of these genetic changes, a series of experiments were performed using muscle samples and genetically modified cell lines. Furthermore, we provide a detailed overview of the clinical and pathological manifestations associated with *TK2* mutations.

## Materials and methods

### Patients

The female patient, born following a full-term pregnancy and vaginal delivery. By 3 months of age, she demonstrated the ability to turn over, and by 5 months, she could sit without support. However, at 8 months, she began to display symptoms of neck muscle weakness, decreased physical endurance, weak crying, and challenges maintaining seated or prone positions without assistance, occasionally exhibiting bucking movements. On physical examination, she exhibited alertness and cognitive acuity. Her cranial nerve functions were within normal limits, with no evidence of ophthalmoplegia, ptosis, or tongue fasciculations. Tendon reflexes were absent. The patient presented with diffuse hypotonia, generalized weakness, and absent tendon reflexes.

By 11 months of age, her spontaneous movements decreased, leading to respiratory dyspnea and the need for mechanical ventilation and nasogastric tube feeding. Laboratory analysis showed elevated venous lactate levels of 3.8 mmol/L (reference range: 1.5–2.99 mmol/L) and serum creatine kinase (CK) levels of 2157 U/L. Brain MRI results were within normal limits. Electromyography (EMG) demonstrated myogenic alterations, while metabolic analysis showed heightened levels of urine organic acid and serum acylcarnitine, notably elevated 3-hydroxybutyrate. Unfortunately, at 13 months of age, she experienced severe respiratory distress, ultimately resulting in her passing. Informed consent for both diagnosis and research participation was obtained from the patient's parents. The study protocol was approved by the ethics committee of Qilu Hospital.

### DNA extraction and library preparation

DNA was extracted from peripheral blood using the MagPure Tissue & Blood DNA LQ Kit (Magen, China). DNA concentrations were quantified using a Qubit fluorometer with the Qubit dsDNA HS Assay Kit (Invitrogen, USA). DNA libraries were prepared using the VAHTS Universal Plus DNA Library Prep Kit for MGI V2 and the VAHTS Dual UMI UDB Adapters Set 1 for MGI (Vazyme, China). Intermediate purification steps were performed with Agencourt AMPure XP beads (Beckman Coulter, USA). The quality and concentration of the libraries were evaluated with the Qubit dsDNA HS Assay Kit.

### Genomic analysis

The DNA libraries were sequenced on the DNBSEQ-T7 platform (MGI, China). After bioinformatics filtering, 2036 mutations were retained for analysis. Mutations were ranked based on clinical phenotypic correlation, and each gene was assessed for phenotypic consistency, inheritance patterns, and pathogenicity. A point mutation in the *TK2* gene (c.311G > A) was identified. Further analysis of the *TK2* gene revealed a large deletion, which was confirmed through BAM plots and additional sample comparisons.

### MLPA and mtDNA deletion analysis

Multiplex Ligation-dependent Probe Amplification (MLPA) was performed using a synthetic probe mix targeting six genes associated with mtDNA depletion syndrome, including *TK2* (MRC-Holland, P089-B1). DNA ligation and amplification were conducted following the manufacturer’s instructions, with results analyzed using Coffalyser.Net software. For mtDNA deletion analysis, regions flanking common deletion sites were amplified using KOD FX Neo polymerase (Toyobo, Japan) with specific primers [16].The PCR products were purified using Agencourt AMPure XP beads and analyzed by 1% agarose gel electrophoresis to confirm product size and detect deletions. Detailed primer sequences are provided in Supplementary Material 1.

### Protein structural analysis of mutations

To examine the structural consequences of the p.Arg104His mutation and the deletion spanning exons 5 to 10 (E5-E10 del) in the TK2 protein, we accessed both wild-type and mutant protein structures from the AlphaFold Protein Structure Database. Utilizing PyMOL, a molecular visualization software, we performed a comparative structural analysis to detect any conformational disparities [17]. The full-length protein, comprising 265 amino acids, was meticulously analyzed to pinpoint the locations of these genetic alterations. Specifically, we compared the wild-type arginine at position 104 with the mutant histidine to observe alterations in local structural dynamics and hydrogen bonding. Additionally, we generated a model to visualize the hypothetical impact of the E5-E10 deletion on the protein structure, focusing on the its potential implications for protein function.

### Histopathological examination

Muscle biopsy samples were collected from the left biceps brachii of the proband. Histopathological examination involved the preparation of serial cryosections measuring 10 μm in thickness. The staining procedures utilized in this study encompassed hematoxylin and eosin (H&E) for overall morphology, modified Gomori trichrome (MGT) for visualization of mitochondrial abnormalities, succinate dehydrogenase (SDH) and cytochrome c oxidase (COX) for mitochondrial activity, as well as SDH-COX double staining to evaluate the functionality of the mitochondrial respiratory chain. Additionally, Oil Red O (ORO) staining was employed to emphasize lipid accumulation.

### Cell culture and transfection procedures

HEK293T cells, sourced from the American Type Culture Collection (ATCC), were maintained in Dulbecco's Modified Eagle Medium (DMEM; Gibco) supplemented with 10% v/v fetal bovine serum (FBS; Gibco, USA) and 100 U/ml penicillin/streptomycin (Sigma, USA) [18]. The cells were incubated in a controlled environment at 37 °C with 5% CO_2_ and adequate humidity. Prior to transfection, cells in 6-well plates were cultured until reaching 60–70% confluence. Transfections were carried out using Lipofectamine 2000 (Invitrogen, L11668019) according to the manufacturer's recommended protocol. Various genetic constructs, including *TK2* siRNA to knock down TK2 expression. Cells were either treated with a scramble plasmid (Scramble), or transfected with *TK2* deletion plasmid (Dele) or *TK2* total plasmid (Total). Following transfection, the cells were incubated at 37 °C with 5% CO_2_ for 48 h to facilitate efficient gene silencing. The sequences of the small interfering siRNA and plasmids can be found in supplementary material 2.

### Western blot analysis

Protein samples ranging from 15 to 30 μg were extracted from muscle homogenates and various transfected cell lines using radioimmunoprecipitation assay (RIPA) lysis buffer (Beyotime, China), supplemented with a combination of protease and phosphatase inhibitors. The Western blotting protocol involved protein electrophoresis, membrane transfer, and blocking procedures, subsequently followed by the application of primary antibodies. The primary antibodies utilized in this study included anti-TK2 (ARP61417_P050, Aviva, USA), OXPHOS cocktail (ab110413, ab110411, Abcam, UK), anti-ATP6 (55313-1-AP, Proteintech, USA), anti-ATP8 (26123-1-AP, Proteintech, USA), anti-CO4 (11242-1-AP, Proteintech, USA), anti-CYB (ab81215, Abcam, UK), anti-ND5 (55410-1-AP, Proteintech, USA), anti-VDAC1 (ab15895, Abcam, UK), anti-CO2 (55070-1-AP, Proteintech, USA), anti-β-actin (ab8226, Abcam, UK), and anti-TUBULIN (A12289, abclonal, China) as loading controls. Detection was performed using Horseradish peroxidase (HRP)-conjugated secondary antibodies, specifically goat anti-mouse IgG and goat anti-rabbit IgG (Jackson). Using a Tanon 5500 camera system, we captured the resulting images of protein bands using Western ECL Substrate (Millipore, USA).

### Quantification of mtDNA copy number

mtDNA copy number was determined using quantitative PCR (qPCR). Total DNA was extracted from both control and patient samples. Relative mtDNA content was assessed by comparing the threshold cycle values of a mitochondrial gene to a nuclear reference gene [19]. The real-time PCR was performed using SYBR qPCR Master Mix (Vazyme, Q712-02/03). Results were analyzed using the ΔΔCt method, normalizing mtDNA quantities to the control, expressed as a percentage. Detailed primer sequences are provided in Supplementary Material 1.

#### Measurement of ROS level

In order to assess reactive oxygen species (ROS) production, cells were collected and reconstituted in phosphate-buffered saline (PBS) at a concentration of 1 × 10^6 cells/ml. Subsequently, the cells were exposed to 5 μM DCFH-DA (Solarbio, CA1410) for a duration of 15 min to facilitate ROS staining [20]. Following staining, the cells underwent thorough washing to eliminate any excess dye. The fluorescence intensity, serving as an indicator of ROS levels, was then quantified through flow cytometry analysis.

#### Quantification of cellular ATP levels

ATP production was quantified using the ATP Assay Kit (Beyotime, S0027). We lysed 1 × 10^6 cells in 200 μL of the provided ATP assay buffer. From this lysate, 10 μL was transferred to a 96-well plate and mixed with 90 μL of ATP reaction mixture. Luminescence was measured following the manufacturer’s protocol to assess ATP synthesis [21]. To accurately determine the ATP concentrations in our samples, we generated a standard curve using ATP standards included in the kit. Results were reported as concentrations of ATP in the samples.

#### Mitochondrial stress test

To examine the impact of genetic variations on mitochondrial function, we conducted mitochondrial stress tests using a Seahorse XFe24 analyzer (Agilent, USA), Seahorse analyzes mitochondrial function by measuring the oxygen consumption rate (OCR) in cells. HEK293T cells from various transfection groups (4 × 10^4 cells each) were plated on an XFe24 plate and allowed to adhere within a CO2 incubator [16, 22]. For the assay, mitochondrial inhibitors were introduced to the culture: 1 μM oligomycin to assess ATP-linked OCR, 1 μM FCCP to determine maximal respiratory capacity, and 0.5 μM each of rotenone and antimycin A to evaluate both basal respiration and proton leak. This protocol facilitates detailed profiling of mitochondrial dynamics in live cells.

#### Mitochondrial respiratory complex assay

To evaluate the performance of individual mitochondrial respiratory chain complexes, we used the Seahorse XFe24 Analyzer, which assesses mitochondrial function by measuring the OCR in cells [22]. HEK293T cells were transitioned from their standard growth medium to a mitochondrial assay solution (MAS) buffer. Cell permeabilization was achieved with 25 μg/ml digitonin to enhance substrate accessibility. The activity of each complex was then sequentially inhibited using specific compounds: 2 μM rotenone, 10 mM succinate, 5 μM antimycin A, 10 mM ascorbate, and 500 μM TMPD. The pH of all solutions and subsequent injections was rigorously maintained at 7.4 to ensure optimal enzymatic function and assay reproducibility.

#### Statistical analysis

Statistical evaluations were performed using GraphPad Prism software (version 9.0). All quantitative data are presented as means ± standard deviations (SDs). Difference among groups were analyzed by two-tailed unpaired Student’s t-test and with a significance threshold of *P* < 0.05. All assays were repeated at least three times.

## Results

### Genetic analysis

**Fig. 1:**
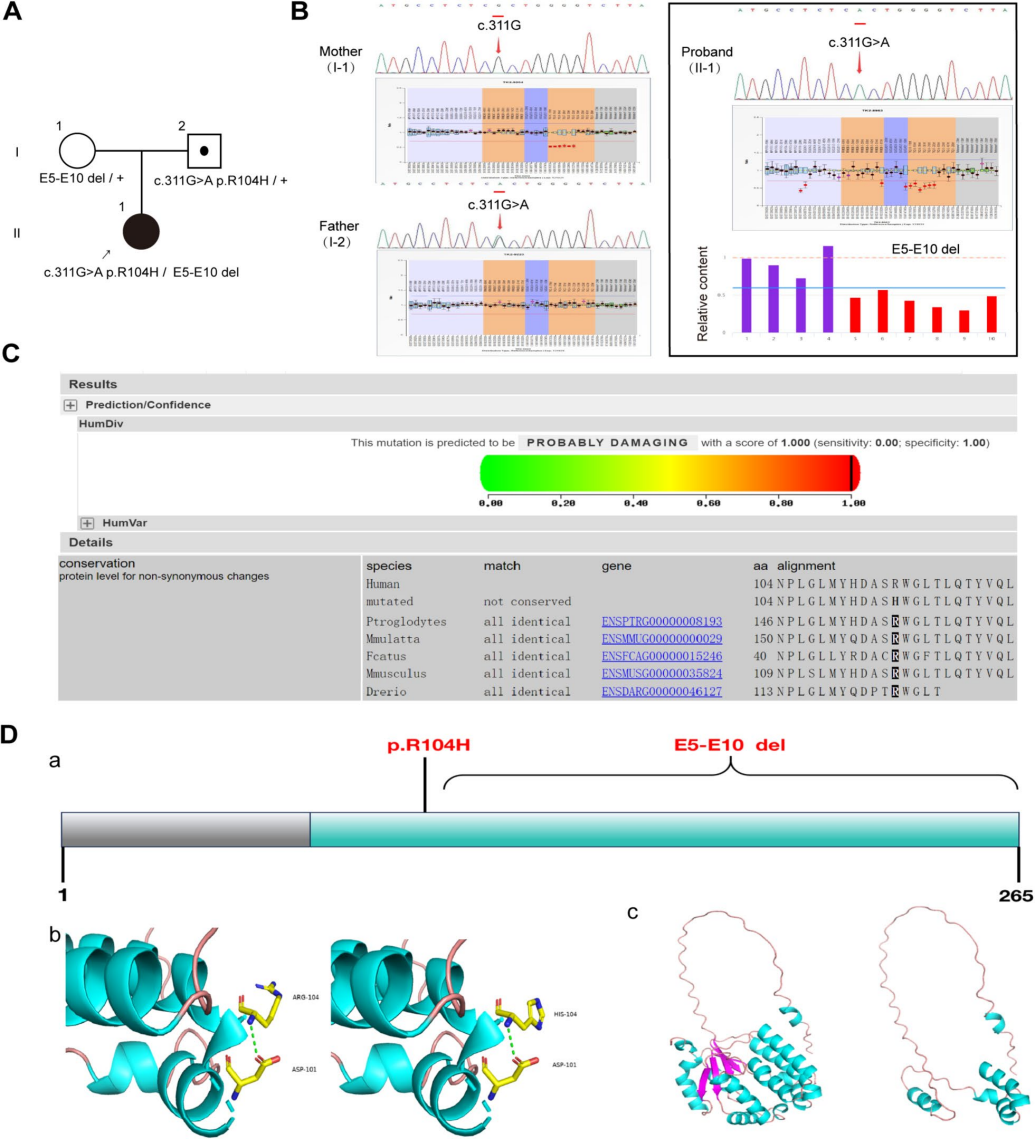
Characterization of the *TK2* compound heterozygous mutations in the patient. **A** Pedigree diagram of the family indicating the inheritance of the *TK2* compound heterozygous mutations. **B** Sanger sequencing chromatograms showing the heterozygous c.311G > A mutation in the proband and the father (I-2). The large deletion mutation (E5–E10 del) in the proband was detected through MLPA, and this mutation was inherited from the patient's mother. **C** In silico prediction of the pathogenicity of the c.311G > A (p.Arg104His) mutation using the PolyPhen-2 software. The missense mutation is predicted to be "probably damaging". **D** Schematic of the TK2 protein structure **a** showing the location of the missense mutation (p.Arg104His) and the large deletion (E5–E10 del) mutation. Structural modeling **b** depicts the predicted impact of the p.Arg104His mutation on protein conformation. Structural modeling **c** shows the severe truncation resulting from the E5–E10 deletion

Whole genome sequencing in the proband revealed compound heterozygous molecular defects in the TK2 gene (NM_004614.5). The first mutation is novel large deletion from exons 5–10 (g.66545871_66565372del), causing a major change in the protein's structure and potentially leading to the loss of functional domains. Through MLPA analysis, we determined that this deletion mutation was inherited from the patient's mother. The second mutation, a missense mutation (c.311G > A) resulting in a p.Arg104His amino acid substitution, was inherited from the father. This missense mutation has been previously reported by Garone et al. [24] (Fig. 1A, B). Based on the variant classification criteria PVS1_Strong, PM2_Supporting, and PP4, the deletion mutation spanning exons 5 to 10 is classified as likely pathogenic. According to the ACMG guidelines, the c.311G > A variant was classified as a likely pathogenic variant (PM1 + PM2_Supporting + PM3 + PP4). Analysis of conservation across various mammalian species indicated that the amino acid residue at position 104 is highly conserved, suggesting its potential importance in protein function (Fig. 1C). Structural predictions suggest that the p.Arg104His substitution could affect protein stability and function by changing its hydrogen bonding network and spatial configuration. The exon 5–10 deletion creates a severely shortened protein lacking important regions for function, potentially causing significant impairment in mitochondrial function (Fig. 1D).

### Histopathological study

**Fig. 2:**
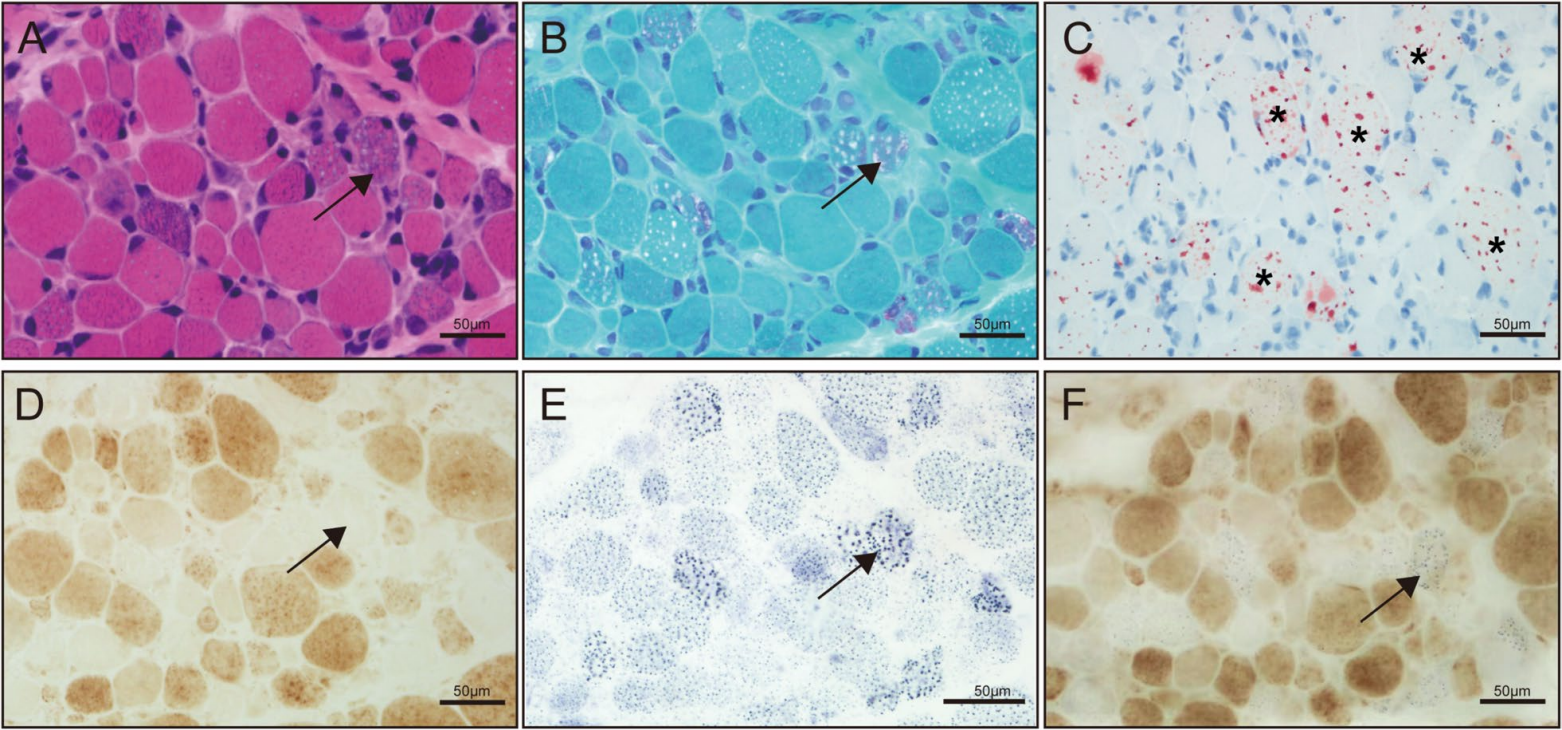
Histopathological characterization of the patient. **A** H&E staining revealed slight variability in muscle fiber size, with dark subsarcolemmal staining in several fibers. **B** MGT staining revealed numerous RRFs (arrow). **C** ORO staining highlights extensive lipid droplet accumulation (asterisks). **D** COX staining demonstrates COX-negative fibers (arrow). **E** SDH staining shows increased SDH activity in many fibers (arrow). **F** Combined COX-SDH staining identifies fibers with both COX deficiency and SDH positivity (arrow). H&E, hematoxylin and eosin; MGT, modified Gomori trichrome; RRFs, ragged red fibers; ORO, Oil Red O COX, cytochrome c oxidase; SDH, succinate dehydrogenase; RBFs, ragged blue fibers. Scale bar: 50 μm

Serial sections of the biceps brachii revealed pathological features consistent with typical metabolic myopathy. Histochemical staining demonstrated significant variability in fiber size, along with numerous COX-negative fibers, which showed enhanced succinate dehydrogenase staining, indicating mitochondrial dysfunction. Additionally, ORO staining revealed a marked accumulation of lipid droplets (Fig. 2).

### Novel biallelic *TK2* mutations reduced the expression levels of TK2 and mitochondrial proteins

**Fig. 3:**
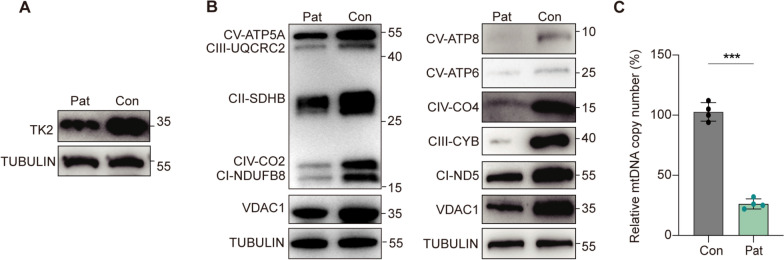
TK2 expression, mitochondrial protein levels, and mtDNA copy number in muscle samples from patient and control. **A** Western blot analysis of TK2 protein levels in muscle samples from the patient (Pat) and control (Con). A significant reduction in TK2 expression is observed in the patient’s muscle. **B** Western blot analysis of mitochondrial respiratory chain complex subunits in muscle samples. The analysis includes subunits of complex I (CI-ND5), complex II (CII-SDHB), complex III (CIII-UQCRC2, CIII-CYB), complex IV (CIV-CO2, CIV-CO4), and complex V (CV-ATP5A, CV-ATP6, CV-ATP8). The expression levels of mitochondrial proteins are significantly lower in the patient’s muscle. **C** Quantification of mtDNA copy number in muscle samples, showing a significant reduction in the patient compared to the control (***p < 0.001)

To evaluate the impact of the patient's biallelic mutations on TK2 protein levels, we conducted Western blot analysis on muscle samples obtained from patients and age- and gender-matched controls. The results demonstrated a significant reduction in total TK2 protein levels in the patient's muscle sample (Fig. 3A). Additionally, we examined the effect of the *TK2* mutations on the synthesis of respiratory chain complex subunits encoded by both nDNA and mtDNA. The patient's muscle biopsy demonstrated a significant reduction in the levels of several essential proteins, such as UQCRC2, CO2, ATP8, ATP6, CO4, CYB, and ND5 (Fig. 3B). Given that *TK2* mutations are linked to MDS, we quantified mtDNA copy number in the muscle samples and found a substantial decrease, with levels dropping to 25% relative to controls (Fig. 3C).

### *TK2* large deletion mutation severely impairs the expression of mitochondrial proteins

**Fig. 4:**
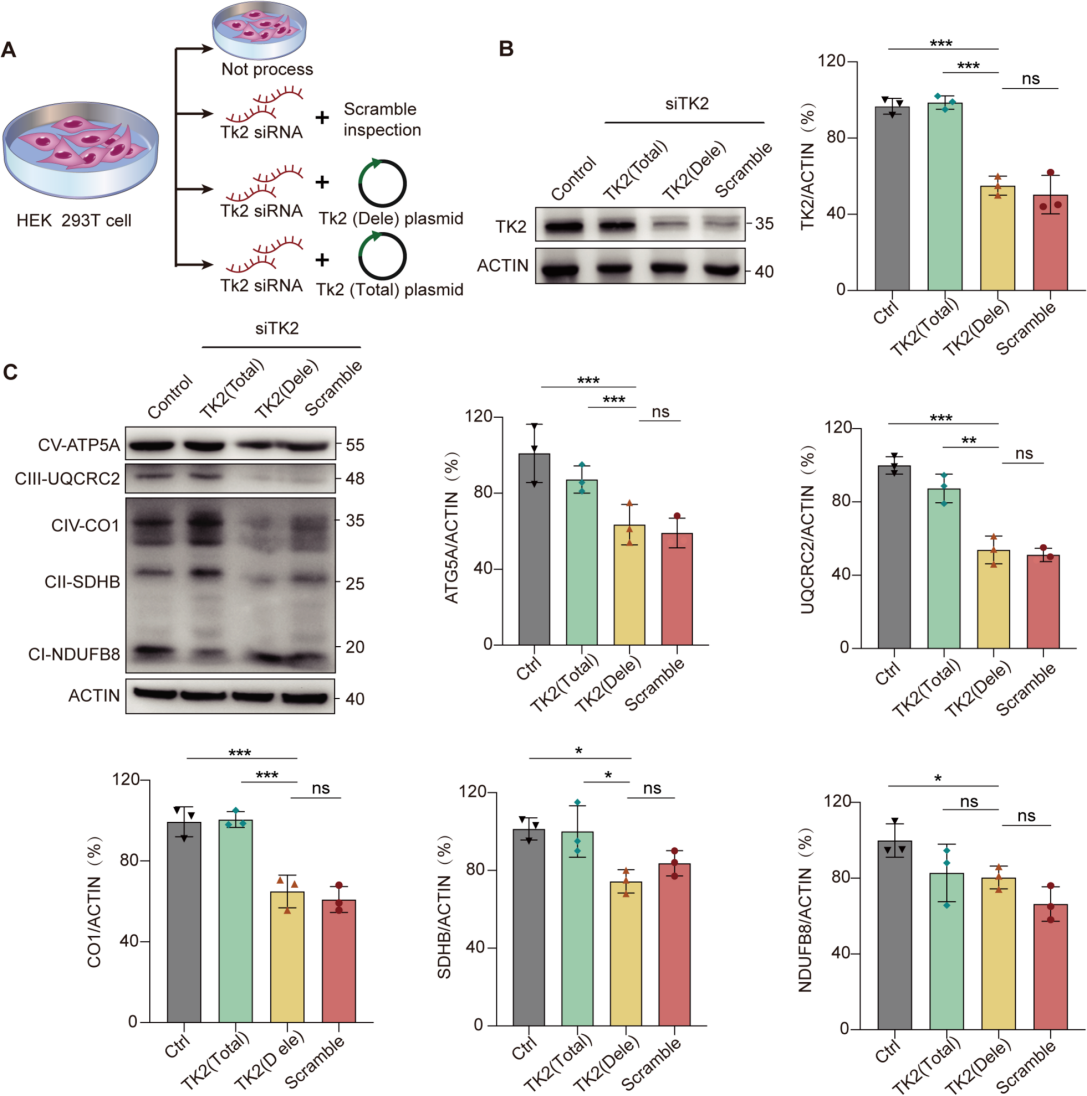
Construction of *TK2* deletion cell model and analysis of TK2 and mitochondrial protein levels. **A** Schematic representation of the experimental design. HEK 293T cells were treated with *TK2* siRNA to knock down TK2 expression. Cells were either left untreated, treated with a scramble siRNA control, or transfected with *TK2* deletion plasmid (Dele) or *TK2* total plasmid (Total). **B** Western blot analysis of TK2 protein levels across the different experimental groups revealed a significant reduction in TK2 expression in the *TK2* (Dele) group compared to the control and *TK2* (Total) groups. **C** Western blot analysis of mitochondrial respiratory chain subunits (CV-ATP5A, CIII-UQCRC2, CIV-CO1) across the different experimental groups revealed a significant reduction in mitochondrial protein levels in the *TK2* (Dele) group compared to the control and *TK2* (Total) groups. The graphs quantify the expression levels of the respective subunits. Statistical significance is indicated by *p < 0.05, **p < 0.01, ***p < 0.001, and non-significant differences are denoted as (ns)

The *TK2* c.311G > A mutation was classified as Likely Pathogenic, whereas the large deletion was designated as a VUS. Since deletion mutations are very rare and the other allele of deletion mutations in most patients is a pathogenic missense mutation, in order to further explore the role of deletion mutations in them, we constructed a *TK2* deletion mutant cell model for research.Using the HEK293T cell line, we generated *TK2* knockdown (Scramble), *TK2* deletion mutants (Dele), and *TK2* knockdown-corrected cells (Total), while also culturing normal HEK293T cells as a control group (Control) (Fig. 4A). Western blot analysis of TK2 protein expression levels across the four cell groups revealed significantly reduced TK2 protein expression in the Dele group, with levels at 53.5% compared to control group and 50.1% compared to Total group (Fig. 4B). Furthermore, we conducted an analysis of the protein levels of mitochondrial respiratory chain complex subunits across different cell groups. Relative to both the Control and the Total group, the Dele group exhibited a marked reduction in the expression of several mitochondrial complex subunit proteins, including ATP5A (61.6%, 79.1%), UQCRC2 (52.2%, 66.3%), CO1 (62.4%, 60.1%) (Fig. 4C).

### *TK2* large deletion mutation contributed to mitochondrial respiration deficiency

**Fig. 5:**
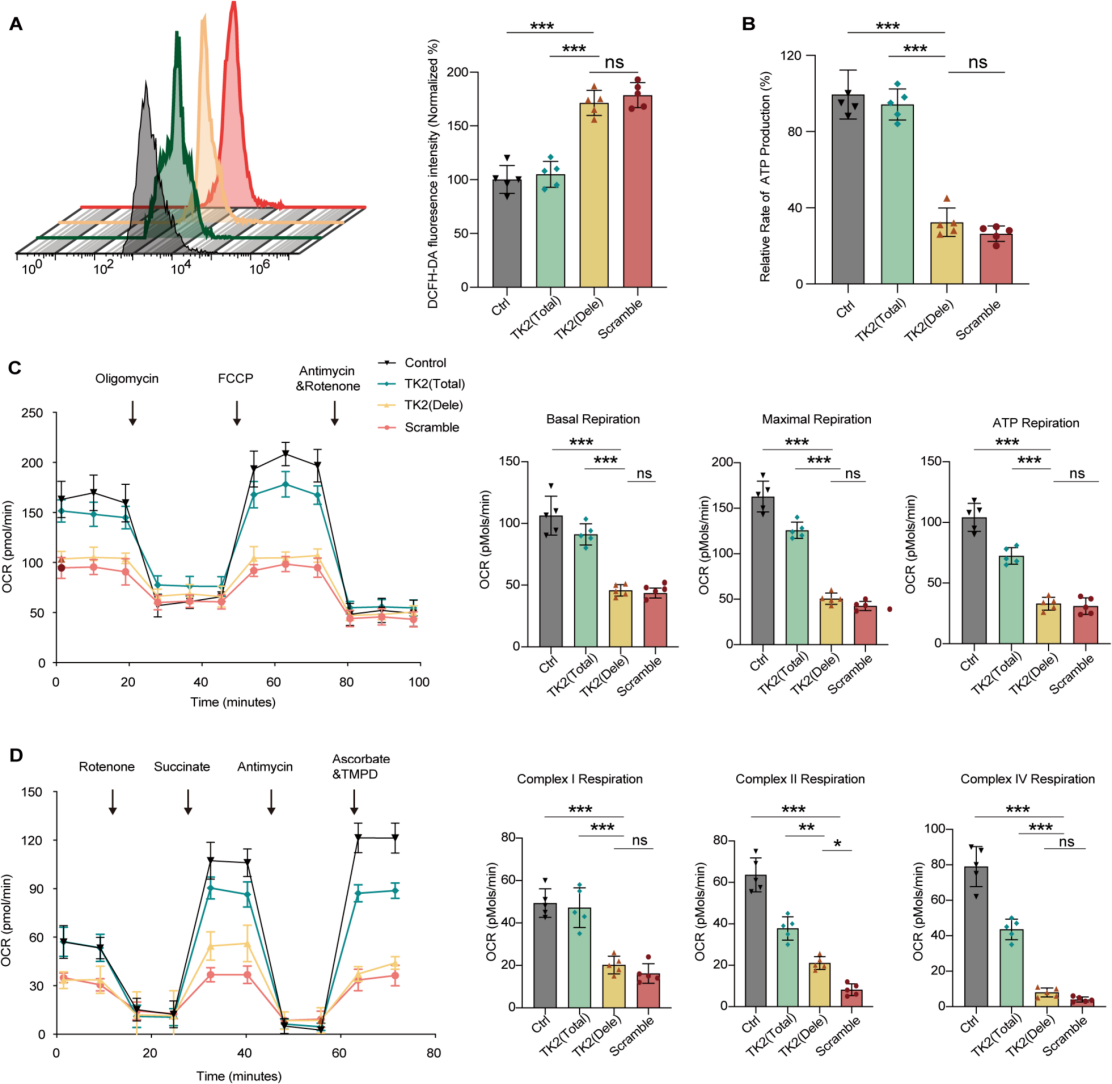
Analysis of ROS levels, ATP production, and mitochondrial respiration in *TK2* deletion cell. **A** Flow cytometry analysis of ROS levels using DCFH-DA fluorescence intensity. The graph on the right shows the quantification of DCFH-DA fluorescence, indicating significantly elevated ROS levels in the *TK2* (Dele) group compared to control and *TK2* (Total) groups. **B** Measurement of ATP production rates in the different experimental groups. The relative rate of ATP production is significantly lower in the *TK2* (Dele) group compared to the control and *TK2* (Total) groups, while no significant difference is observed between the control and scramble groups. **C** OCR analysis using the Seahorse Bioscience XFe 24 Extracellular Flux Analyzer to measure mitochondrial respiration. The graph depicts basal respiration, maximal respiration, and ATP production in the different groups. The *TK2* (Dele) group shows significant reductions in all parameters compared to the control and *TK2* (Total) groups. **D** Analysis of mitochondrial complex-specific respiration (complexes I, II, and IV) using the Seahorse Bioscience XFe 24. The graphs show reduced activity in all complexes in the *TK2* (Dele) group compared to the control and *TK2* (Total) groups. Statistical significance is represented as *p < 0.05, **p < 0.01, ***p < 0.001, and non-significant differences as (ns)

Reactive oxygen species (ROS) are well-recognized byproducts generated during energy-demanding conditions, often considered harmful reactive molecules that can damage cellular structures. In this study, ROS levels were assessed using DCFH-DA fluorescence intensity, which was significantly elevated in the Dele group compared to the Control (177.2%) and Total (169.6%) groups (Fig. 5A). Additionally, ATP production was markedly reduced in the Dele group, compared to the Control (31.5%) and Total (35.7%) groups (Fig. 5B). We further analyzed the OCR in these four cell groups. Basal respiration (47.3%, 55.7%), maximum respiration (29.4%, 41.6%), and ATP-linked OCR (39.7%, 49.8%) in Dele cells were significantly lower compared to the Control and Total cells (Fig. 5C). To investigate the site-specific effects of this mutation on mitochondrial respiratory chain (MRC) activity, we measured complex I-, II-, and IV-mediated respiration. Our results revealed that Dele group exhibited marked reductions in respiration across all complexes (I 40.6%, 43.8%, II 32.3%, 51.3%, and IV 12.5%, 23.9%) compared to the Control and Total groups (Fig. 5D).

### The spectrum of *TK2* mutations

**Fig. 6:**
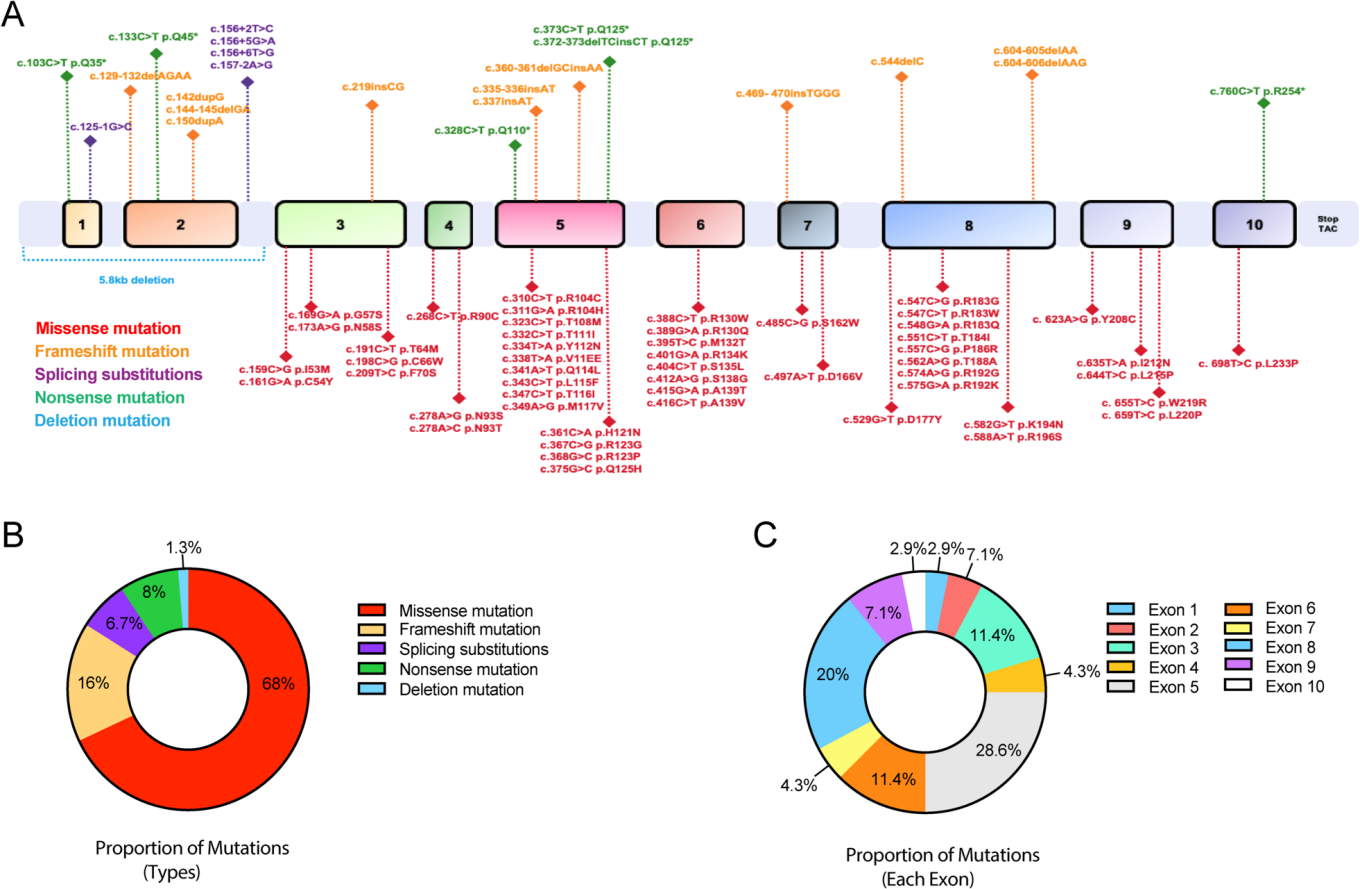
Mutation Spectrum of the *TK2* Gene. **A** Schematic representation of the *TK2* gene structure. Mutations are color-coded according to type: missense mutations (red), frameshift mutations (green), splicing substitutions (purple), nonsense mutations (orange), and deletion mutations (blue). The diagram visualizes the mutation landscape. **B** Pie chart showing the proportion of different types of mutations in the *TK2* gene. Missense mutations are the most common, followed by frameshift, splicing substitutions, nonsense, and deletion mutations. **C** Pie chart illustrating the proportion of mutations across each exon of the *TK2* gene. Exons 5 and 8 contain the highest percentage of mutations

The *TK2* gene is located on chromosome 16, with an mRNA length of 5114 nucleotides, including a 3.8-kilobase pair 3′ UTR region, and consists of 10 exons (NM_004614.5) [23]. To provide a thorough depiction of the distribution and categorization of mutation sites within the gene, we have precisely mapped 75 distinct reported mutation sites onto the gene's structural framework (Fig. 6A). Among these, missense mutations are the most frequently observed, with 51 sites identified. Conversely, reports of large deletion mutations are extremely rare, with only one mutation currently documented. Furthermore, the documented mutation sites are predominantly clustered in exons 5 and 8 (Fig. 6B, C).

To further investigate the clinical features and mutation characteristics of patients with *TK2* gene mutations, we reviewed 250 reported cases. We excluded 200 patients due to incomplete data on onset time and clinical presentation. Ultimately, 50 cases with relatively complete patient data were selected for further analysis. These cases included 29 male and 21 female patients from diverse ethnic backgrounds. Based on age of onset, patients were categorized into three groups: early-onset (≤ 1 year, 15 cases), childhood-onset (> 1–12 years, 26 cases), and late-onset (> 12 years, 9 cases). Among these 50 cases, nearly half experienced respiratory failure, and almost all exhibited elevated CK levels. Muscle biopsy results show that approximately 60% of patients exhibit muscle fibers with both RRF and COX deficiency, while around 20% of patients display lipid accumulation. However, in patients with *TK2* mutations, increased lipid droplet accumulation in muscle has often been associated with poorer prognosis. The most frequent *TK2* mutations were c.323C > T (p.Thr108Met), present in 18 alleles, and c.361C > A (p.His121Asn), present in 7 alleles. Further analysis revealed that missense mutations were the most common type, comprising 76% of the mutations, followed by frameshift mutations (12%), nonsense mutations (6%), and splice site mutations (4%). Deletion mutations were the rarest, accounting for only 1% (Table 1).

**Table 1:** Clinical, pathological, and genetical characteristics of patients with *TK2* mutations

Patient	Gender	Ethnicity	Age at diagnose	Age of death/alive	Clinical findings				Histochemical findings		
					Ptosis	Limb-girdle muscle weakness	Respiratory failure	Facial muscle weakness	RRF	COX-	Lipid accumulation
1	M	Latino	3.5 yr	Alive	+	+	−	+	+	−	+
2	F	Indian	46 yr	Alive	−	+	−	+	+	+	−
3	M	Hispanic	2.8 yr	4.75 yr	−	−	+	−	+	−	−
4	M	Hispanic	1.5 yr	Alive	−	−	+	−	−	−	−
5	F	Hispanic	14 yr	Alive	−	−	+	−	−	−	+
6	F	NA	13mo	22mo	−	−	−	−	−	+	−
7	M	Swedish	18mo	3.5 years	−	+	+	−	+	+	+
8	F	Japanese	Birth	10 months	−	+	+	−	NA	+	+
9	M	NA	22 yr	Alive	−	+	+	+	NA	+	−
10	M	NA	2mo	10mo	−	−	−	−	+	+	−
11	F	Spanish	6mo	Alive	−	+	−	+	+	+	−
12	F	NA	74 yr	Alive	+	+	+	+	NA	+	+
13	M	Sephardic-Jewish	3.5 yr	Alive	−	+	−	−	+	NA	−
14	M	Ashkenazi Jewish	24mo	16 yr	+	−	+	−	+	+	−
15	F	African American	24mo	6 yr	−	−	+	−	+	+	−
16	M	Brazilian	7mo	19 yr	−	+	+	−	+	+	−
17	F	Chilean	24mo	Alive	−	+	−	−	+	+	−
18	F	NA	Birth	NA	+	+	−	−	+	+	−
19	F	NA	12 yr	NA	+	−	−	+	+	+	−
20	M	NA	50 yr	NA	+	−	−	+	+	+	−
21	F	NA	23 yr	NA	−	−	−	+	+	+	−
22	M	NA	15 yr	NA	+	−	−	+	+	+	−
23	F	NA	12 yr	NA	+	−	−	+	NA	NA	−
24	F	NA	30 yr	NA	+	−	−	+	+	+	−
25	M	Chinese	10mo	2 yr	−	+	+	−	+	+	−
26	M	Chinese	12mo	2 yr	−	+	+	−	+	+	−
27	F	Chinese	1 yr	2 yr	−	+	+	−	+	+	−
28	M	NA	14mo	Alive	−	−	−	−	+	+	−
29	M	NA	16 yr	Alive	−	−	−	+	+	+	−
30	F	NA	23 yr	43 yr	−	−	+	+	+	+	+
31	F	Italian	8 yr	NA	+	+	−	+	+	+	−
32	M	Latin American	9mo	NA	−	−	+	−	+	+	−
33	F	Latin American	9 yr	NA	−	−	+	−	+	−	−
34	M	Caucasian	2 yr	NA	+	−	+	−	+	+	−
35	M	Latin American	Birth	NA	+	−	+	−	+	+	−
36	M	Caucasian	4 yr	NA	−	−	+	−	+	+	−
37	M	Caucasian	5mo	3 yr	−	−	−	−	+	−	−
38	M	Chinese	2 yr	3.5 yr	−	−	+	−	+	+	−
39	F	NA	0.08 yr	2 yr	−	−	−	−	NA	NA	NA
40	M	NA	0.5 yr	6 yr	+	−	−	−	NA	NA	NA
41	M	NA	0.92 yr	NA	−	−	−	−	NA	NA	NA
42	M	NA	0.83 yr	1.5 yr	−	−	−	−	NA	NA	NA
43	M	NA	1.25 yr	NA	−	−	−	−	NA	NA	NA
44	F	NA	2 yr	NA	−	−	−	+	NA	NA	NA
45	M	NA	3 yr	NA	+	−	−	−	NA	NA	NA
46	M	NA	1.08 yr	1.5 yr	−	−	−	−	NA	NA	NA
47	M	NA	1.17 yr	NA	−	−	−	−	NA	NA	NA
48	M	NA	4.42 yr	NA	−	−	−	−	NA	NA	NA
49	F	NA	4 yr	NA	+	−	−	+	NA	NA	NA
50	F	Chinese	8mo	13mo	−	+	+	−	+	+	+

Patient	Laboratory examination			*TK2* mutation1(Exon)	Mutation type	*TK2* mutation 2 (Exon)	Mutation type	MtDNA content	References
	Elevated serum CK	EMG	Respiratory chain enzyme activity						
1	+	Myopathic	NA	c.361C > A, p.His121Asn(5)	Missense	c.361C > A, p.His121Asn (5)	Missense	NA	Wang et al., (2018) [23]
2	+	NA	NA	c.469-470insTGGG(7)	Frameshift	c.156 + 6 T > G(intron)	Splicing substitutions	Low level	Wang et al., (2018) [23]
3	+	Myopathic	NA	c.173A > G, p.Asn58Ser(3)	Missense	c.173A > G, p.Asn58Ser (3)	Missense	NA	Wang et al., (2018) [23]
4	+	NA	NA	c.361C > A, p.His121Asn (5)	Missense	c.361C > A, p.His121Asn (5)	Missense	NA	Wang et al., (2018) [23]
5	+	NA	NA	c.604_606delAAG p.Lys202del(8)	Frameshift	c.575G > A p.Arg192Lys(8)	Missense	NA	Wang et al., (2018) [23]
6	+	NA	Complex I (63%), Complex IV (66%)	C.416C > T p.Ala139Val(6)	Missense	C.209 T > C p.Phe70Ser(3)	Missense	NA	Mazurova et al., (2017) [37]
7	+	Normal	NA	c.156 + 5G > C(intron)	Splicing substitutions	c.332C > T p.Thr111Ile(5)	Missense	29%	Roos et al., (2014) [38]
8	+	NA	Complex I, III, IV and V markedly decreased	c.125-1G > C(1)	Splicing substitutions	c.574A > G, p.Arg192Gly(8)	Missense	8.0%	Termglinchan et al., (2016) [7]
9	+	NA	NA	c.323C > T p.Thr108Met(5)	Missense	c.323C > T p.Thr108Met (5)	Missense	55.0%	Paradas et al., (2013) [39]
10	NA	NA	Complex IV decreased	C.547C > G p.Arg183Gly(8)	Missense	C.760C > T p.Arg254*(10)	Nonsense	60.0%	Carrozzo et al., (2003) [40]
11	+	Myopathic	Complex III decreased	c.323C > T p.Thr108Met (5)	Missense	c.323C > T p.Thr108Met (5)	Missense	18.0%	Martín-Hernández et al., (2017) [41]
12	NA	NA	NA	c.103C > T, p.Gln35*(1)	Nonsense	c.582 G > T p.Lys194Asn(8)	Missense	185%	Alston et al., (2013) [42]
13	−	NA	Complex I and I + III reduction	C.268C > T p.His90Asn(4)	Missense	C.268C > T p.His90Asn (4)	Missense	NA	Leshinsky-Silver et al., (2008) [43]
14	+	Myopathic	NA	c.635 T > A, p.Thr212Asn(9)	Missense	AT ins 337(5)	Frameshift	NA	Oskoui et al., (2006) [26]
15	+	Myopathic	NA	c.360G > A p.Arg120Arg (5)	Synonymous	c.361C > A, p.His121Asn (5)	Missense	NA	Oskoui et al., (2006) [26]
16	+	NA	NA	c.323C > T p.Thr108Met (5)	Missense	c.372_373delTCinsCT, p.Gln125X(5)	Nonsense	NA	Oskoui et al., (2006) [26]
17	+	Neurogenic	NA	c.278A > G p.Asn93Ser(4)	Missense	AT ins 337(5)	Frameshift	NA	Oskoui et al., (2006) [26]
18	+	Myopathic	Complex I, III, IV decreased	c.323C > T p.Thr108Met (5)	Missense	c.323C > T p.Thr108Met (5)	Missense	NA	Béhin et al., (2012) [44]
19	+	NA	Complex I, III and IV deficit	c.323C > T p.Thr108Met (5)	Missense	c.323C > T p.Thr108Met (5)	Missense	17.0%	Domínguez-González et al., (2019) [45]
20	+	NA	Normal	c.604–606 AAGdel p.Lys202del(8)	Frameshift	c.604–606 AAGdel p.Lys202del(8)	Frameshift	66.0%	Domínguez-González et al., (2019) [45]
21	−	NA	Complex I, III and IV deficit	c.323C > T p.Thr108Met (5)	Missense	c.323C > T p.Thr108Met(5)	Missense	35.0%	Domínguez-González et al., (2019) [45]
22	+	NA	Complex I, III and IV deficit	c.623A > G p.Tyr208Cys(9)	Missense	c.623A > G p.Tyr208Cys(9)	Missense	NA	Domínguez-González et al., (2019) [45]
23	+	NA	NA	c.623A > G p.Tyr208Cys(9)	Missense	c.623A > G p.Tyr208Cys(9)	Missense	NA	Domínguez-González et al., (2019) [45]
24	+	NA	Normal	c.323C > T p.Thr108Met 5)	Missense	c.323C > T p.Thr108Met(5)	Missense	53.0%	Domínguez-González et al., (2019) [45]
25	+	Myopathic	NA	c.144_145del p.Glu48fs(2)	Frameshift	c.547C > T p.Arg183Trp(8)	Missense	NA	Hu et al., (2020) [46]
26	+	Normal	NA	c.659 T > C p.Leu220Pro(9)	Missense	c.497A > T p.Asp166Val(7)	Missense	NA	Hu et al., (2020) [46]
27	+	NA	NA	c.497A > T p.Asp166Val(7)	Missense	c.328C > T p.Gln110*(5)	Nonsense	NA	Hu et al., (2020) [46]
28	+	NA	Complex I, III and IV deficit	c.416C > T, p.Ala139Val (6)	Missense	c.416C > T, p.Ala139Val(6)	Missense	NA	Papadimas et al., (2020) [47]
29	+	NA	Normal	c.323C > T p.Thr108Met (5)	Missense	c.323C > T p.Thr108Met(5)	Missense	< 30.0%	de Fuenmayor-Fernández de la Hoz et al., (2021) [48]
30	NA	NA	Complex I, III and IV deficit	c.323C > T, p.Thr108Met (5)	Missense	c.323C > T, p.Thr108Met (5)	Missense	NA	Laine-Menéndez et al., (2021) [49]
31	+	NA	NA	c.278A > G, p.Asn93Ser (4)	Missense	c.543del, p.Leu182Phefs ∗ 11(8)	Frameshift	30.0%	Manini et al., (2022) [50]
32	+	NA	NA	c.547C > T p.Arg183Trp(8)	Missense	c.547C > T p.Arg183Trp(8)	Missense	25.0%	Manini et al., (2022) [50]
33	NA	NA	NA	c.173A > G p.Asn58Ser(3)	Missense	c.173A > G p.Asn58Ser(3)	Missense	86.0%	Manini et al., (2022) [50]
34	+	NA	NA	c.389G > A p.Arg130Gln(6)	Missense	c.129_132delAGAA(2)	Frameshift	24.0%	Manini et al., (2022) [50]
35	+	NA	NA	c.361C > A p.His121Asn (5)	Missense	c.361C > A p.His121Asn (5)	Missense	72.0%	Manini et al., (2022) [50]
36	+	NA	NA	C.157-2A > G, 157minus2AtoG(intron)	Splicing substitutions	c.588A > T p.Arg196Ser(8)	Missense	73.0%	Manini et al., (2022) [50]
37	+	NA	NA	c.323C > T; p.Thr108Met (5)	Missense	C.698 T > C p. Leu233Pro(10)	Missense	5.0%	Manini et al., (2022) [50]
38	+	NA	NA	c.659 T > C p.Leu220Pro(8)	Missense	c.161G > A p. Cys54Tyr(3)	Missense	NA	Wu et al., (2018) [51]
39	NA	NA	Complex I, II + III, I + III, IV decreased	c.159C > G p.Ile53Met(3)	Missense	c.159C > G p.Ile53Met(3)	Missense	14.0%	Garone et al., (2018) [24]
40	NA	Myopathic	NA	c.198C > G p.Cys66Trp;(3)	Missense	c.644 T > C p.Leu215Pro(9)	Missense	8.4%	Garone et al., (2018) [24]
41	NA	NA	Complex II + III, IV decreased	C.311C > A p.Arg104His(5)	Missense	c.388C > T p.Arg130Trp6)	Missense	31.0%	Garone et al., (2018) [24]
42	+	NA	NA	c.372_373delTCinsCT p.Gly125X(5)	Nonsense	C.395 T > C p.Met132Thr(6)	Missense	10.0%	Garone et al., (2018) [24]
43	+	NA	Complex I, IV decreased	c.159C > G p.Ile53Met(3)	Missense	c.159C > G p.Ile53Met(3)	Missense	14.0%	Garone et al., (2018) [24]
44	+	Neurogenic	Complex II + III, IV,decreased	C.335_336dup p.Val113Metfs20*;(5)	Frameshift	c.278A > G p.Asn93Ser(4)	Missense	21.0%	Garone et al., (2018) [24]
45	+	Myopathic	NA	c.198C > G p.Cys66Trp;(3)	Missense	c.644 T > C p.Leu215Pro(9)	Missense	9.2%	Garone et al., (2018) [24]
46	+	NA	Complex I, IV decreased	c.133C > T p.Gln45* (2)	Nonsense	c.173A > G p.Asn58Ser(3)	Missense	5.0%	Garone et al., (2018) [24]
47	+	Myopathic	Complex I, III, IV decreased	c.360_361delinsAA p.His121Trp;(5)	Frameshift	c.575G > A p.Arg192Lys (8)	Missense	17.0%	Garone et al., (2018) [24]
48	+	NA	NA	c.150dup p.Ser51fs;(2)	Frameshift	c.375G > C p.Gln125His(5)	Missense	24.0%	Garone et al., (2018) [24]
49	+	Myopathic	Complex I, III, IV decreased	c.347C > T p.Thr116Ile;(5)	Missense	c.347C > T p.Thr116Ile(5)	Missense	31.0%	Garone et al., (2018) [24]
50	+	Myopathic	Complex I, II, IV decreased	E5-E10 del	Deletion	c.311C > A p.Arg104His(5)	Missense	23.4%	This study

*F* female; *M* male; *mo* month; *yr* year; *CK* creatinine kinase; *RRF* ragged red fiber; *COX* cytochrome c oxidase; *EMG* electromyography; *NA* not available

## Discussion

MDS is a group of rare autosomal recessive mitochondrial disorders first described in 1991. These disorders are characterized by a significant reduction in mtDNA content, leading to a variety of clinical manifestations that can affect multiple organ systems, including skeletal muscle, liver, and brain. *TK2*-related MDS is particularly rare, with only around 60 cases reported globally since its first description in 2001. Moreover, skeletal muscle has a significantly higher demand for mtDNA-encoded proteins compared to other tissues, such as the liver and brain, yet exhibits relatively low TK2 activity [2]. This discrepancy may explain why *TK2*-related diseases predominantly manifest in skeletal muscle.

In this study, we describe a case involving a neonate diagnosed with MDS, characterized by two compound heterozygous mutations in the *TK2* gene: a large deletion spanning exons 5–10, and a missense mutation, c.311G > A (p.Arg104His). The missense mutation, has been previously documented by Garone et al. [24]. Several lines of evidence suggest that the novel compound heterozygous mutations are pathogenic. First, the patient exhibited clinical symptoms and pathological features consistent with early-onset mitochondrial disease. Second, these genetic defects co-segregated with the observed phenotype in the affected family. Third, a significant reduction in mtDNA copy number was identified in muscle samples from the patient. Fourth, the compound heterozygous mutations resulted in decreased expression of MRC complexes, with a particularly pronounced effect on the expression levels of subunits from complexes I, III, and IV.

Clinically, TK2 deficiency is characterized by a range of symptoms, including hypotonia, muscle weakness, ptosis, and exercise intolerance, which are typical of mitochondrial myopathies [9, 25–27]. Besides presenting as mitochondrial myopathy, *TK2* mutations may also be associated with other clinical manifestations, including sensorineural hearing loss, PEO/PMM with multiple mtDNA deletions, early-onset encephalopathy, SMA-like phenotype, and multisystemic involvement [28–30]. In our case, the patient presented with severe hypotonia, consistent with the classical clinical features of TK2 deficiency. It is worth noting that our patient developed symptoms as early as infancy, and the patient's condition deteriorated rapidly and died within 5 months after onset. Considering the in-frame deletion of exons 5–10 in the *TK2* gene in our patient, we hypothesize that this leads to the production of a truncated protein. The truncated protein lacks essential functional domains, which likely severely impacts its biological function. This may be a major contributing factor to the severity of the clinical symptoms observed.

Genotypic analysis of 50 patients revealed a high incidence of the p.Thr108Met mutation, with a broad range of disease onset: 4 cases were early-onset, 2 were childhood-onset, and 4 were late-onset. Disease severity varied among patients, which is consistent with the findings of Ceballos et al. [13]. Additionally, in the analysis of 8 late-onset cases, we identified 2 patients with the p. Lys202del mutation, which was exclusive to the late-onset group. One patient was heterozygous for p.Thr192Lys, while the other had a homozygous p.Lys202del mutation. Both patients exhibited normal electromyography and respiratory chain enzyme activity, and did not experience severe infantile disease, suggesting that p.Lys202del is a mild variant, as reported by Wang et al. [23].

Previous studies have focused on point mutations and small deletions, limiting our understanding of the full spectrum of *TK2* mutations in MDS. For example, the p.Lys202del mutation, which results in an in-frame deletion, is associated with a mild clinical phenotype, characterized by delayed onset and slow progression [13]. In contrast, we identified a significant 29.9 kb deletion in the *TK2* gene of an infant, spanning exons 5–10. The breakpoints of this deletion lie within introns, causing an in-frame loss of the coding sequence. As this deletion also includes the 3' UTR region, it disrupts normal transcription. Unlike the late-onset pattern seen in p.Lys202del cases, the infant exhibited early onset, severe symptoms, and rapid disease progression.

Additionally, in our muscle histology study, we observed a marked increase in lipid droplets, which suggests a possible disruption in fatty acid metabolism. A similar observation of lipid accumulation in muscle biopsy was reported by Marti in a Spanish case with a *TK2* mutation [30]. Mitochondrial function is critical for the development and differentiation of adipose tissue, and there is evidence that alterations in mtDNA can disrupt adipose tissue metabolism [31]. An in vivo study on *Tk2*^−/−^ mice also revealed significant changes in mitochondrial proteins, respiratory complex activities, and genes associated with lipid metabolism [32]. Furthermore, MRC disorders, have previously been reported in a patient with lipid accumulation, suggesting impaired mitochondrial fatty acid oxidation [33]. In our patient, the extensive lipid droplet deposition was a key observation, and we also noted a decline in MRC function, which initially led us to consider a lipid storage disorder.

Currently, there are no effective treatments for MDS caused by *TK2* mutations. However, nucleoside-based therapy, which involves supplementation with exogenous deoxycytidine(dCtd) and dThd, represents a promising experimental approach for *TK2* deficiency. Studies in *Tk2H126N* knock-in mice treated with oral dCMP and dTMP extended their lifespan by 2–3 times [34]. Further research in *Tk2*^−/−^ mice revealed that skeletal muscle is the primary target of dCtd + dThd therapy [35]. Additionally, a clinical study involving 16 patients with TK2 deficiency demonstrated that nucleoside therapy could reverse early-onset quadriplegia, halt mechanical ventilation, and stop percutaneous endoscopic gastrostomy (PEG), resulting in significant functional improvement. Fifteen of these patients, who received oral dCtd + dThd, showed remarkable clinical benefits [36]. These findings highlight the critical importance of early genetic diagnosis and the timely initiation of deoxynucleoside therapy, which can improve respiratory muscle function and significantly extend the survival of MDS patients associated with TK2 mutations.

In summary, this study revealed the effect of a new compound heterozygous mutation of the *TK2* gene on mitochondrial function, focusing on the large fragment deletion mutation sites that have never been reported, and verified its pathogenicity. In addition, our study enriches the *TK2* gene lineage and expands the understanding of mitochondrial myopathy caused by *TK2* gene mutation, which lays a foundation for further understanding of the disease and proposing possible improved diagnosis and treatment methods in the future.

## Supplementary Information

Additional file 1.

Additional file 2.

Additional file 3.

## Data Availability

All data relevant to the study are included in the article and in and its supplementary materials. Any reagents used within this study can be obtained from the corresponding author upon a reasonable request.

## References

[1] Rahman S, Copeland WC. POLG-related disorders and their neurological manifestations. Nat Rev Neurol. 2019;15:40–52. 30451971 10.1038/s41582-018-0101-0 PMC8796686

[2] Pérez-Pérez M-J, Priego E-M, Hernández A-I, Familiar O, Camarasa M-J, Negri A, et al. Structure, physiological role, and specific inhibitors of human thymidine kinase 2 (TK2): present and future. Med Res Rev. 2008;28:797–820. 18459168 10.1002/med.20124 PMC7168489

[3] Bourdon A, Minai L, Serre V, Jais J-P, Sarzi E, Aubert S, et al. Mutation of RRM2B, encoding p53-controlled ribonucleotide reductase (p53R2), causes severe mitochondrial DNA depletion. Nat Genet. 2007;39:776–80. 17486094 10.1038/ng2040

[4] Götz A, Isohanni P, Pihko H, Paetau A, Herva R, Saarenpää-Heikkilä O, et al. Thymidine kinase 2 defects can cause multi-tissue mtDNA depletion syndrome. Brain. 2008;131:2841–50. 18819985 10.1093/brain/awn236

[5] El-Hattab AW, Scaglia F. Mitochondrial DNA depletion syndromes: review and updates of genetic basis, manifestations, and therapeutic options. Neurotherapeutics. 2013;10:186–98. 23385875 10.1007/s13311-013-0177-6 PMC3625391

[6] Ganguly BB, Kadam NN. Mutations of myelodysplastic syndromes (MDS): an update. Mutat Res Rev Mutat Res. 2016;769:47–62. 27543316 10.1016/j.mrrev.2016.04.009

[7] Termglinchan T, Hisamatsu S, Ohmori J, Suzumura H, Sumitomo N, Imataka G, et al. Novel TK2 mutations as a cause of delayed muscle maturation in mtDNA depletion syndrome. Neurol Genet. 2016;2:e95. 27660820 10.1212/NXG.0000000000000095 PMC5024793

[8] Chanprasert S, Wang J, Weng S-W, Enns GM, Boué DR, Wong BL, et al. Molecular and clinical characterization of the myopathic form of mitochondrial DNA depletion syndrome caused by mutations in the thymidine kinase (TK2) gene. Mol Genet Metab. 2013;110:153–61. 23932787 10.1016/j.ymgme.2013.07.009

[9] Tulinius M, Moslemi A-R, Darin N, Holme E, Oldfors A. Novel mutations in the thymidine kinase 2 gene (TK2) associated with fatal mitochondrial myopathy and mitochondrial DNA depletion. Neuromuscul Disord. 2005;15:412–5. 15907288 10.1016/j.nmd.2005.03.010

[10] Bitter EE, Townsend MH, Erickson R, Allen C, O’Neill KL. Thymidine kinase 1 through the ages: a comprehensive review. Cell Biosci. 2020;10:138. 33292474 10.1186/s13578-020-00493-1 PMC7694900

[11] Berardo A, Domínguez-González C, Engelstad K, Hirano M. Advances in thymidine kinase 2 deficiency: clinical aspects, translational progress, and emerging therapies. J Neuromuscul Dis. 2022;9:225–35. 35094997 10.3233/JND-210786 PMC9028656

[12] Domínguez-González C, Madruga-Garrido M, Hirano M, Martí I, Martín MA, Munell F, et al. Collaborative model for diagnosis and treatment of very rare diseases: experience in Spain with thymidine kinase 2 deficiency. Orphanet J Rare Dis. 2021;16:407. 34600563 10.1186/s13023-021-02030-w PMC8487573

[13] Ceballos F, Serrano-Lorenzo P, Bermejo-Guerrero L, Blázquez A, Quesada-Espinosa JF, Amigo J, et al. Clinical and genetic analysis of patients with TK2 deficiency. Neurol Genet. 2024;10:e200138. 38544965 10.1212/NXG.0000000000200138 PMC10965359

[14] Munch-Petersen B. Enzymatic regulation of cytosolic thymidine kinase 1 and mitochondrial thymidine kinase 2: a mini review. Nucleosides Nucleotides Nucleic Acids. 2010;29:363–9. 20544521 10.1080/15257771003729591

[15] Desler C, Munch-Petersen B, Rasmussen LJ. The role of mitochondrial dNTP levels in cells with reduced TK2 activity. Nucleosides Nucleotides Nucleic Acids. 2006;25:1171–5. 17065084 10.1080/15257770600894501

[16] Qian W, Kumar N, Roginskaya V, Fouquerel E, Opresko PL, Shiva S, et al. Chemoptogenetic damage to mitochondria causes rapid telomere dysfunction. Proc Natl Acad Sci USA. 2019;116:18435–44. 31451640 10.1073/pnas.1910574116 PMC6744920

[17] Lu X-J. DSSR-enabled innovative schematics of 3D nucleic acid structures with PyMOL. Nucleic Acids Res. 2020;48:e74. 32442277 10.1093/nar/gkaa426 PMC7367123

[18] Adikusuma F, Lushington C, Arudkumar J, Godahewa GI, Chey YCJ, Gierus L, et al. Optimized nickase- and nuclease-based prime editing in human and mouse cells. Nucleic Acids Res. 2021;49:10785–95. 34534334 10.1093/nar/gkab792 PMC8501948

[19] Livak KJ, Schmittgen TD. Analysis of relative gene expression data using real-time quantitative PCR and the 2(-Delta Delta C(T)) Method. Methods. 2001;25:402–8. 11846609 10.1006/meth.2001.1262

[20] Jiang M, Jike Y, Liu K, Gan F, Zhang K, Xie M, et al. Exosome-mediated miR-144-3p promotes ferroptosis to inhibit osteosarcoma proliferation, migration, and invasion through regulating ZEB1. Mol Cancer. 2023;22:113. 37461104 10.1186/s12943-023-01804-z PMC10351131

[21] Liu Y, Lu Y, Ning B, Su X, Yang B, Dong H, et al. Intravenous delivery of living listeria monocytogenes elicits gasdmermin-dependent tumor pyroptosis and motivates anti-tumor immune response. ACS Nano. 2022;16:4102–15. 35262333 10.1021/acsnano.1c09818

[22] Zhao X, Cui L, Xiao Y, Mao Q, Aishanjiang M, Kong W, et al. Hypertension-associated mitochondrial DNA 4401A>G mutation caused the aberrant processing of tRNAMet, all 8 tRNAs and ND6 mRNA in the light-strand transcript. Nucleic Acids Res. 2019;47:10340–56. 31504769 10.1093/nar/gkz742 PMC6821173

[23] Wang J, Kim E, Dai H, Stefans V, Vogel H, Al Jasmi F, et al. Clinical and molecular spectrum of thymidine kinase 2-related mtDNA maintenance defect. Mol Genet Metab. 2018;124:124–30. 29735374 10.1016/j.ymgme.2018.04.012

[24] Garone C, Taylor RW, Nascimento A, Poulton J, Fratter C, Domínguez-González C, et al. Retrospective natural history of thymidine kinase 2 deficiency. J Med Genet. 2018;55:515–21. 29602790 10.1136/jmedgenet-2017-105012 PMC6073909

[25] Zhang J, Frerman FE, Kim J-JP. Structure of electron transfer flavoprotein-ubiquinone oxidoreductase and electron transfer to the mitochondrial ubiquinone pool. Proceed Natl Acad Sci. 2006;103(44):16212–7. 10.1073/pnas.0604567103. 10.1073/pnas.0604567103 PMC1637562 17050691

[26] Oskoui M, Davidzon G, Pascual J, Erazo R, Gurgel-Giannetti J, Krishna S, et al. Clinical spectrum of mitochondrial DNA depletion due to mutations in the thymidine kinase 2 gene. Arch Neurol. 2006;63:1122–6. 16908738 10.1001/archneur.63.8.1122

[27] Blakely E, He L, Gardner JL, Hudson G, Walter J, Hughes I, et al. Novel mutations in the TK2 gene associated with fatal mitochondrial DNA depletion myopathy. Neuromuscul Disord. 2008;18:557–60. 18508266 10.1016/j.nmd.2008.04.014

[28] Lesko N, Naess K, Wibom R, Solaroli N, Nennesmo I, von Döbeln U, et al. Two novel mutations in thymidine kinase-2 cause early onset fatal encephalomyopathy and severe mtDNA depletion. Neuromuscul Disord. 2010;20:198–203. 20083405 10.1016/j.nmd.2009.11.013

[29] Pons R, Andreetta F, Wang CH, Vu TH, Bonilla E, DiMauro S, et al. Mitochondrial myopathy simulating spinal muscular atrophy. Pediatr Neurol. 1996;15:153–8. 8888051 10.1016/0887-8994(96)00118-x

[30] Martí R, Nascimento A, Colomer J, Lara MC, López-Gallardo E, Ruiz-Pesini E, et al. Hearing loss in a patient with the myopathic form of mitochondrial DNA depletion syndrome and a novel mutation in the TK2 gene. Pediatr Res. 2010;68:151–4. 20421844 10.1203/PDR.0b013e3181e33bbe

[31] Villarroya J, Giralt M, Villarroya F. Mitochondrial DNA: an up-and-coming actor in white adipose tissue pathophysiology. Obesity (Silver Spring). 2009;17:1814–20. 19461585 10.1038/oby.2009.152

[32] Villarroya J, Cereijo R, Gavaldà-Navarro A, Peyrou M, Giralt M, Villarroya F. New insights into the secretory functions of brown adipose tissue. J Endocrinol. 2019;243:R19-27. 31419785 10.1530/JOE-19-0295

[33] Watmough NJ, Bindoff LA, Birch-Machin MA, Jackson S, Bartlett K, Ragan CI, Poulton J, Gardiner RM, Sherratt HS, Turnbull DM. Impaired mitochondrial beta-oxidation in a patient with an abnormality of the respiratory chain. Studies in skeletal muscle mitochondria. J Clin Invest. 1990;85(1):177–84. 10.1172/JCI114409. 2153151 10.1172/JCI114409 PMC296403

[34] Lopez-Gomez C, Levy RJ, Sanchez-Quintero MJ, Juanola-Falgarona M, Barca E, Garcia-Diaz B, et al. Deoxycytidine and deoxythymidine treatment for thymidine kinase 2 deficiency. Ann Neurol. 2017;81:641–52. 28318037 10.1002/ana.24922 PMC5926768

[35] Lopez-Gomez C, Hewan H, Sierra C, Akman HO, Sanchez-Quintero MJ, Juanola-Falgarona M, et al. Bioavailability and cytosolic kinases modulate response to deoxynucleoside therapy in TK2 deficiency. EBioMedicine. 2019;46:356–67. 31383553 10.1016/j.ebiom.2019.07.037 PMC6710986

[36] Domínguez-González C, Madruga-Garrido M, Mavillard F, Garone C, Aguirre-Rodríguez FJ, Donati MA, et al. Deoxynucleoside therapy for thymidine kinase 2-deficient myopathy. Ann Neurol. 2019;86:293–303. 31125140 10.1002/ana.25506 PMC7586249

[37] Mazurova S, Magner M, Kucerova-Vidrova V, Vondrackova A, Stranecky V, Pristoupilova A, et al. Thymidine kinase 2 and alanyl-tRNA synthetase 2 deficiencies cause lethal mitochondrial cardiomyopathy: case reports and review of the literature. Cardiol Young. 2017;27:936–44. 27839525 10.1017/S1047951116001876

[38] Roos S, Lindgren U, Ehrstedt C, Moslemi AR, Oldfors A. Mitochondrial DNA depletion in single fibers in a patient with novel TK2 mutations. Neuromuscul Disord. 2014;24:713–20. 24953930 10.1016/j.nmd.2014.05.009

[39] Paradas C, Gutiérrez Ríos P, Rivas E, Carbonell P, Hirano M, DiMauro S. TK2 mutation presenting as indolent myopathy. Neurology. 2013;80:504–6. 23303857 10.1212/WNL.0b013e31827f0ff7 PMC3590052

[40] Carrozzo R, Bornstein B, Lucioli S, Campos Y, de la Pena P, Petit N, et al. Mutation analysis in 16 patients with mtDNA depletion. Hum Mutat. 2003;21:453–4. 12655576 10.1002/humu.9135

[41] Martín-Hernández E, García-Silva MT, Quijada-Fraile P, Rodríguez-García ME, Rivera H, Hernández-Laín A, et al. Myopathic mtDNA depletion syndrome due to mutation in TK2 gene. Pediatr Dev Pathol. 2017;20:416–20. 28812460 10.1177/1093526616686439

[42] Alston CL, Schaefer AM, Raman P, Solaroli N, Krishnan KJ, Blakely EL, et al. Late-onset respiratory failure due to TK2 mutations causing multiple mtDNA deletions. Neurology. 2013;81:2051–3. 24198295 10.1212/01.wnl.0000436931.94291.e6 PMC3854830

[43] Leshinsky-Silver E, Michelson M, Cohen S, Ginsberg M, Sadeh M, Barash V, et al. A defect in the thymidine kinase 2 gene causing isolated mitochondrial myopathy without mtDNA depletion. Eur J Paediatr Neurol. 2008;12:309–13. 17951082 10.1016/j.ejpn.2007.09.005

[44] Béhin A, Jardel C, Claeys KG, Fagart J, Louha M, Romero NB, et al. Adult cases of mitochondrial DNA depletion due to TK2 defect: an expanding spectrum. Neurology. 2012;78:644–8. 22345218 10.1212/WNL.0b013e318248df2b

[45] Domínguez-González C, Hernández-Laín A, Rivas E, Hernández-Voth A, Sayas Catalán J, Fernández-Torrón R, et al. Late-onset thymidine kinase 2 deficiency: a review of 18 cases. Orphanet J Rare Dis. 2019;14:100. 31060578 10.1186/s13023-019-1071-z PMC6501326

[46] Hu C, Li X, Zhao L, Shi Y, Zhou S, Wang Y. Clinical profile and outcome of pediatric mitochondrial myopathy in China. Front Neurol. 2020;11:1000. 33013660 10.3389/fneur.2020.01000 PMC7506116

[47] Papadimas GK, Vargiami E, Dragoumi P, Van Coster R, Smet J, Seneca S, et al. Mild myopathic phenotype in a patient with homozygous c.416C > T mutation in TK2 gene. Acta Myol. 2020;39:94–7. 32904881 10.36185/2532-1900-012 PMC7460728

[48] Pablo C, Morís G, Jiménez-Mallebrera C, Badosa C, Hernández-Laín A, Encinar AB, Martín MÁ, Domínguez-González C. Recurrent rhabdomyolysis and exercise intolerance: a new phenotype of late-onset thymidine kinase 2 deficiency. Mol Genet Metab Rep. 2021;26:100701. 10.1016/j.ymgmr.2020.100701. 33457207 10.1016/j.ymgmr.2020.100701 PMC7797901

[49] Laine-Menéndez S, Domínguez-González C, Blázquez A, Delmiro A, García-Consuegra I, Fernández-de la Torre M, et al. Preferent diaphragmatic involvement in TK2 deficiency: an autopsy case study. Int J Mol Sci. 2021;22:5598. 34070501 10.3390/ijms22115598 PMC8199166

[50] Manini A, Meneri M, Rodolico C, Corti S, Toscano A, Comi GP, et al. Case report: thymidine kinase 2 (TK2) deficiency: a novel mutation associated with childhood-onset mitochondrial myopathy and atypical progression. Front Neurol. 2022;13:857279. 35280287 10.3389/fneur.2022.857279 PMC8914305

[51] Wu Y, Liu DH, Zhang XX. Thymidine kinase 2 gene compound heterozygous mutation leads to mitochondrial DNA depletion syndrome-2:a case report. Zhonghua Er Ke Za Zhi. 2018;56:381–2. 29783828 10.3760/cma.j.issn.0578-1310.2018.05.016

