# Supplementary material for: Novel biallelic TK2 mutations cause mitochondrial DNA depletion syndrome with infantile early-onset lipid storage myopathy
Source: Orphanet J Rare Dis. 2025 Mar 17;20:130. doi: 10.1186/s13023-025-03639-x (PMC11912596; doi:10.1186/s13023-025-03639-x)
Supplement: Supplementary file 1 — Additional file 1. [file 13023_2025_3639_MOESM1_ESM.docx]

Supplementary Material 1. The detailed primer sequences used in this study.

| MT-ND1 | Forward: 5’-ATGGCCAACCTCCTACTCCT-3’ |
| --- | --- |
|  | Reverse: 5’-GCGGTGATGTAGAGGGTGAT-3’ |
| MT-ND4 | Forward: 5’-CCTGACTCCTACCCCTCACA-3’ |
|  | Reverse: 5’-GAAGTATGTGCCTGCGTTCA-3’ |
| MT-ND5 | Forward: 5’-AACTGTTCATCGGCTGAGAG-3’ |
|  | Reverse: 5’-GCTAGGAGGAGGCCTAGTAG-3’ |
| MT-RNR1 | Forward: 5’-GCTAAACCTAGCCCCAAACC-3’ |
|  | Reverse: 5’-TTGGCTCTCCTTGCAAAGTT-3’ |

| MT-ND1 | Forward: 5’-ATGGCCAACCTCCTACTCCT-3’ |
| --- | --- |
|  | Reverse: 5’-GCGGTGATGTAGAGGGTGAT-3’ |
| MT-ND4 | Forward: 5’-CCTGACTCCTACCCCTCACA-3’ |
|  | Reverse: 5’-GAAGTATGTGCCTGCGTTCA-3’ |
| MT-ND5 | Forward: 5’-AACTGTTCATCGGCTGAGAG-3’ |
|  | Reverse: 5’-GCTAGGAGGAGGCCTAGTAG-3’ |
| MT-RNR1 | Forward: 5’-GCTAAACCTAGCCCCAAACC-3’ |
|  | Reverse: 5’-TTGGCTCTCCTTGCAAAGTT-3’ |
| GAPDH | Forward: 5’-GGAGTCCACTGGCGTCTTCA -3’ |
|  | Reverse: 5’-GTCATGAGTCCTTCCACGATACC -3’ |
| MT-CO3 | Forward: 5’-CAGCCCATGACCCCTAACAG-3’ |
|  | Reverse: 5’-TACATCGCGCCATCATTGGT-3’ |
