# Supplementary material for: Novel biallelic TK2 mutations cause mitochondrial DNA depletion syndrome with infantile early-onset lipid storage myopathy
Source: Orphanet J Rare Dis. 2025 Mar 17;20:130. doi: 10.1186/s13023-025-03639-x (PMC11912596; doi:10.1186/s13023-025-03639-x)
Supplement: Supplementary file 2 — Additional file 2. [file 13023_2025_3639_MOESM2_ESM.docx]

**Supplementary Material 2: TK2 siRNA and plasmid construction information.**

**TK2 siRNA sequence information**

Sense strand: 5'-GACCAAUCCUGAGACUUGUTT-3'

Antisense strand: 5'-ACAAGUCUCAGGAUUGGUCTT-3'

**Plasmid 1: pGV658-TK2 (Dele)**

- **Plasmid Name**: pGV658-TK2 (Dele)
- **Vector Backbone**: GV658
- **Key Elements**: CMV enhancer, Multiple Cloning Site (MCS), polyA, EF1A promoter driving zsGreen, SV40 promoter controlling puromycin resistance.
- **Cloning Sites**: KpnI / PacI
- **PCR Primers**:

**Forward Primer (TK2-p1)**:

5'-GTGGATCCGAGCTCGGTACCCGCCACCATGCTGCTGTGGCCGCTGCG-3'

**Reverse Primer (TK2-p2)**:

5'-ATATTTTATTACCGGTTTAATTAACTACAGAGGATTGTGGCCACGGAC-3'

- **Verification**: PCR product size of 339 bp. Positive clones confirmed by sequencing.

**Plasmid 2: pGV658-Control (Scramble)**

- **Plasmid Name**: pGV658-Control (Scramble)
- **Vector Backbone**: GV658
- **Key Elements**: Same as above (CMV enhancer, MCS, polyA, EF1A-zsGreen, SV40-puromycin).
- **Cloning Sites**: KpnI / PacI
- Control Function: This plasmid serves as the negative control for the experiments involving the overexpression of TK2. It contains the same backbone and selection markers as the experimental plasmids but without the TK2 gene.

**Plasmid 3: pGV658-TK2 (Total)**

- **Plasmid Name**: pGV658-TK2 (Total)
- **Vector Backbone**: GV658
- **Key Elements**: Same as above (CMV enhancer, MCS, polyA, EF1A-zsGreen, SV40-puromycin).
- **Cloning Sites**: KpnI / PacI
- **PCR Primers**:

**Forward Primer (TK2-p1)**:

5'-GTGGATCCGAGCTCGGTACCCGCCACCATGCTGCTGTGGCCGCTGCG-3'

**Reverse Primer (TK2-p2)**:

5'-ATATTTTATTACCGGTTTAATTAACTATGGGCAATGCTTCCGATTCTC-3'

- **Verification**: PCR product size of 849 bp, positive clones confirmed by sequencing
