# Supplementary figures and images for: Novel biallelic TK2 mutations cause mitochondrial DNA depletion syndrome with infantile early-onset lipid storage myopathy
Source: Orphanet J Rare Dis. 2025 Mar 17;20:130. doi: 10.1186/s13023-025-03639-x (PMC11912596; doi:10.1186/s13023-025-03639-x)

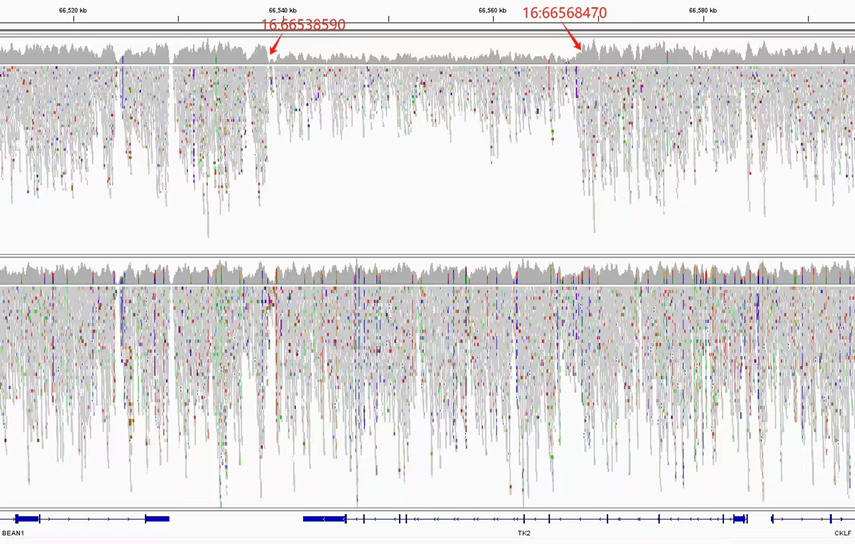

Supplement: Supplementary file 3 — Additional file 3. [file 13023_2025_3639_MOESM3_ESM.docx]
